# A Novel LiDAR Data Classification Algorithm Combined CapsNet with ResNet

**DOI:** 10.3390/s20041151

**Published:** 2020-02-19

**Authors:** Aili Wang, Minhui Wang, Haibin Wu, Kaiyuan Jiang, Yuji Iwahori

**Affiliations:** 1 The Higher Educational Key Laboratory for Measuring & Control Technology and Instrumentations of Heilongjiang, Harbin University of Science and Technology, Harbin 150080, China; aili925@hrbust.edu.cn (A.W.); minhuijy@163.com (M.W.); jiangkaiyuan@hrbust.edu.cn (K.J.); 2 Department of Computer Science, Chubu University, Aichi 487-8501, Japan; iwahori@isc.chubu.ac.jp

**Keywords:** image classification, deep learning, convolutional neural network (CNN), residual network (ResNet), capsule network (CapsNet)

## Abstract

LiDAR data contain feature information such as the height and shape of the ground target and play an important role for land classification. The effect of convolutional neural network (CNN) for feature extraction on LiDAR data is very significant, however CNN cannot resolve the spatial relationship of features adequately. The capsule network (CapsNet) can identify the spatial variations of features and is widely used in supervised learning. In this article, the CapsNet is combined with the residual network (ResNet) to design a deep network-ResCapNet for improving the accuracy of LiDAR classification. The capsule network represents the features by vectors, which can account for the direction of the features and the relative position between the features. Therefore, more detailed feature information can be extracted. ResNet protects the integrity of information by passing input information to the output directly, which can solve the problem of network degradation caused by information loss in the traditional CNN propagation process to a certain extent. Two different LiDAR data sets and several classic machine learning algorithms are used for comparative experiments. The experimental results show that ResCapNet proposed in this article `improve the performance of LiDAR classification.

## 1. Introduction

LiDAR launched in the 1980s and successfully detected the lunar surface for the American Apollo mission to the moon. Because of its huge technical potential, many research scholars have studied it to promote the development continuously and progress of theory and technology. Thus, it becomes an indispensable detection technology in the field of science and technology. LiDAR has many advantages, such as high resolution, good concealment, and strong anti-interference ability. It is widely used in many different fields. For example, it can elevate the measure accuracy of projects that are difficult to measure in construction engineering [1]; it can build the 3D models for historical buildings to record information in terms of cultural relics; it can detect underwater distances to provide data for environmental protection programs [2]; it also can be used to detect landslides and other disasters [3]. In recent years, deep learning has developed rapidly and has achieved remarkable results in various fields [4,5,6,7]. Therefore, this article also uses deep learning algorithms for pixel-level classification of LiDAR data.

The data used in this article are the LiDAR-derived rasterized Digital Surface Models (LiDAR-DSM), which were obtained by processing the points cloud data acquired from the airborne LiDAR system by denoising and rasterization [8]. LiDAR-DSM mainly includes the terrain change of the target area and the feature height of the target object in the area, which is suitable for distinguishing classification tasks with different height targets and measuring planning. It plays an important role in the process for the measurement, planning, and construction of cities [9].

In recent years, the convolutional neural network (CNN) has been introduced into the LiDAR data classification [10], which solves the problem of the parameters to be difficult to adjust and laborious caused by the traditional manual extraction of LiDAR-DSM features. Accurate classification of DSM data plays an important role in distinguishing different feature categories. The classification task of this data is usually based on pixel classification; that is, the interpretation process of remote sensing images [11]. 

At present, there are many studies on LiDAR classification. In 2006, Lodha et al. used Support Vector Machine (SVM) to classify the DSM data, which obtained higher accuracy and convincing visual results [12]. In 2012, Sasaki et al. used decision tree to each land category for analyzing the average height to achieve classification [13]. Naidoo et al. used automated random forest model to classify eight common savanna species [14]. In 2015, Khodadadzadeh et al. developed a new efficient classification strategy for hyperspectral and DSM fusion, integrating multiple types of features and achieving better classification results [15]. In 2016, Ghamisi et al. proposed a method of using DSM data as extended attribute for joint classification with CNN to improve classification accuracy [16]. In 2017, Ghamisi et al. proposed a method to extract spatial and background information of DSM data in an unsupervised manner to obtain higher classification accuracy [17]. In 2018, Wang et al. combined morphology (MPs) and CNN to provide more feature information for DSM classification [10]. Subsequently, He et al. used spatial transformer networks (STN) for identifying the best input image of CNN for LiDAR classification [18]. Xia et al. combined hyperspectral image (HSI) and DSM by using integrated classifiers to process morphological features and classify them [19]. In 2019, Ge et al. proposed a new framework for fusion of HSI and LiDAR data based on the extinction profiles, local binary pattern (LBP), and kernel collaborative representation classification [20]. Wang et al. used spatial transformation network(STN) and densely connected convolutional network (DenseNet) are combined to form STN-DenseNet, which makes the input data adaptively deform according to the network needs, making full use of all information from the front layers of the network [21]. Subsequently, Wang et al. used the Fire modules of SqueezeNet to replace the traditional convolution layers in OctConv to form a new dual neural architecture: OctSqueezeNet, which improved the accuracy and efficiency of the network simultaneously [22].

However, CNN uses scalar to represent the information in many image processing fields. It is difficult for CNN to identify the features when the spatial location of feature information changes. It needs to deepen the layers of network constantly to extract more information [23,24,25,26,27,28,29,30,31]. The capsule network (CapsNet) represents the feature information by a vector, and it can represent the positional relationship between different features and the direction of the feature information. When the same target occurs in position or angle change, it can still be identified accurately by CapsNet [32].

In recent years, CapsNet has been used in many image applications fields. In 2018, Wang et al. proposed a hybrid method based on CapsNet and triple generative adversarial network (TripleGAN) to avoid overfitting and extract the effective features [33]. Ahmad et al. proposed a new architecture for 3D object classification, which is an extension of the Capsule Network to 3D data [34]. In 2019, Zhu et al. proposed a deep capsule network for HSI classification to improve the performance of the CNNs [35]. Paoletti et al. proposed a new CNN architecture based on spectral–spatial capsule networks in order to achieve a highly accurate classification of HIS while reducing the network design complexity [36]; Afshar et al. proposed a modified CapsNet architecture for brain tumor classification, which takes the tumor coarse boundaries as extra inputs within its pipeline to increase the CapsNet’s focus [37]; Yin et al. proposed an alternative data-driven HSI classification model based on CapsNet [38]; Wang et al. proposed a Caps-TripleGAN framework for sample generation and integrated CapsNet for hyperspectral image classification [39].

In addition, for the traditional CNN, with the depth of the network increasing, the performance of network may degrade; that is, when the accuracy of training tends to be flat, the training error becomes larger. Residual network (ResNet) [40] was proposed to solve the problem. ResNet establishes a bypass connection and sends the input to the output directly to avoid the loss of information and to mitigate the degradation of the network. ResNet has significant benefits in many areas. In 2018, Mou et al. propose a novel network architecture, fully Conv–Deconv network, for unsupervised spectral–spatial feature learning of hyperspectral images, which is able to be trained in an end-to-end manner [41]. In the same year, Zhong et al. designed an end-to-end spectral–spatial residual network (SSRN) that takes raw 3-D cubes as input data without feature engineering for hyperspectral image classification [42]; Qin et al. proposed a deep residual neural network based on leukocyte classifier constructed at first, which can imitate the domain expert’s cell recognition process, and extract salient features robustly and automatically [43]. In 2019, Paolett et al. presented a new deep CNN architecture specially designed for the HSI data. A new model pursues to improve the spectral–spatial features uncovered by the convolutional filters of the network [44]. Zhan et al. proposed an attention residual learning convolutional neural network (ARL-CNN) model for skin lesion classification in dermoscopy images, which is composed of multiple ARL blocks, a global average pooling layer, and a classification layer [45].

We combine the advantages of ResNet and CapsNet to design the ResCapNet to obtain more detailed information of LiDAR data for classification applications. The main contributions of this article are as follows.

(1) Combine the CapsNet and ResNet to form a new network framework named ResCapNet. The input features are extracted using ResNet and the outputs of ResNet are sent to CapsNet for further classification.
(2) The proposed method is tested on two different LiDAR data sets to predict for each pixel the land type associated with that pixel while the number of training samples is limited.

The organization of this article is as follows. Section 2 and Section 3 present the CapsNet and ResNet, respectively. Section 4 is dedicated to the details of the proposed classification method in this article and Section 5 reports the experimental results and analysis. Section 6 is the conclusions of the proposed framework.

## 2. Capsule Network

The CapsNet is made up of capsules rather than neurons. A capsule is a small group of neurons that can examine a particular object, such as a rectangle, and learns from a certain area of the feature maps. The output of CapsNet is an n-dimensional vector. The length of each vector represents the estimated probability of the existence of the object and the direction of each vector records the attitude parameters of object, such as the exact position, rotation, thickness, inclination, and size of the object. If the object changes slightly, such as moving, rotating, or changing the size, the CapsNet will obtain an output vector of the same length but with a slight change in direction. Therefore, the feature extraction of CapsNet is not affected by the changes of space for features. Traditional CNNs require additional components to identify each detail of the objects automatically, and CapsNet can represent the hierarchical structure of each detail part directly. CapsNet has two main characteristics: The first is layer-based compression, and the second is dynamic routing.

### 2.1. Layer-Based Compression

**Figure 1:**
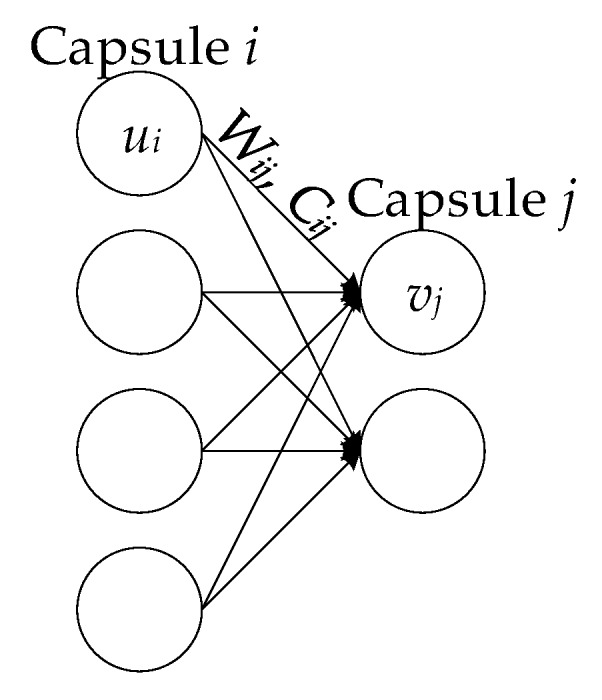
Calculation chart of Capsule.

As shown in Figure 1, both input $u_{i}$ and output $v_{j}$ are vectors. Multiply the transformation matrix $W_{ij}$ with the output $u_{i}$ of the previous capsule for turning the $u_{i}$ to ${\hat{u}}_{j|i}$. Then, in Equation (1) and Equation (2), calculate the weighted sum $s_{i}$ according to the weight $C_{ij}$. $C_{ij}$ is the coupling coefficient, which is calculated through the iteration of dynamic routing process, and specifies the sum of $\sum_{j}c_{ij}$ is 1. $C_{ij}$ measures how likely can capsule i activate capsule j.

(1) $${\hat{u}}_{j|i}=W_{ij}u_{i}$$

(2) $$s_{j}=\sum_{i}c_{ij}{\hat{u}}_{j|i}$$

The activation function of $s_{j}$ is squash instead of ReLU, so the length of the final output vector $v_{j}$ of the capsule is between 0 and 1. This function compresses small vectors to zero and large vectors to unit vectors. The activation function Squash is shown as Equation (3).

(3) $$v_{j}=\frac{{\left\|s_{j}\right\|}^{2}}{1+{\left\|s_{j}\right\|}^{2}}\frac{s_{j}}{\left\|s_{j}\right\|}$$

### 2.2. Dynamic Routing

Capsule calculates the output by calculating the intermediate value $C_{ij}$ through the iterative dynamic routing. In Equation (1) and Equation (2), the prediction vector ${\hat{u}}_{j|i}$ is the prediction (vote) from capsule i and has an impact on the output of capsule j. If the activation vector has a high similarity with the prediction vector, the two capsules are highly correlated. This similarity is measured by the scalar product of the prediction vector and the activation vector.

Therefore, in Equation (4), the similarity score $b_{ij}$ will consider both the possibility of feature existence and the attribute of the feature, unlike neurons, which only consider the possibility of feature existence. At the same time, if the activation $u_{i}$ of the capsule i is very low, since the length of ${\hat{u}}_{j|i}$ is proportional to $u_{i}$, $b_{ij}$ will still be low; that is, if the capsule of the detail feature is not activated, the correlation between the detail feature and the overall feature is very low. The coupling coefficient $C_{ij}$ is calculated by the softmax of $b_{ij}$ in Equation (5):

(4) $$b_{ij}\leftarrow {\hat{u}}_{j|i}\times v_{j}$$

(5) $$C_{ij}\leftarrow \frac{\exp(b_{ij})}{\sum_{k}\exp(b_{ik})}$$

The process of dynamic routing is shown in Algorithm 1 as follows:
**Algorithm 1 Dynamic Routing**Routing (${\hat{u}}_{j|i}$, r, l) **for** all capsule i in layer l-1 and j in layer l: $b_{ij}\leftarrow 0$ **for**
r iterations do   **for** all capsule i in layer l-1: $C_{i}\leftarrow \mathrm{softmax}(b_{i})$   **for** all capsule j in layer l: $s_{j}\leftarrow \sum_{i}c_{ij}{\hat{u}}_{j|i}$   **for** all capsule j in layer l: $v_{j}=\mathrm{squash}(s_{j})$   **for** all capsule i in layer l-1 and j in layer l: $b_{ij}\leftarrow b_{ij}+{\hat{u}}_{j|i}\cdot v_{j}$ **return**
$v_{j}$


Dynamic routing is not a complete replacement for backpropagation. The transformation matrix $W_{ij}$ is still trained by backpropagation, while the dynamic path is only used to calculate the output of the capsule. Calculate the $C_{ij}$ to quantify the connection between the child capsule and its parent capsule. Each data point is re-initialized to 0 before performing dynamic routing calculations [43].

## 3. Residual Network

Deep convolutional networks integrate the characteristics of different levels, such as global features and detail features. The levels of features can be enriched by deepening the network. Therefore, a deeper network structure is used to obtain more detail features generally. However, there is a problem of degradation on traditional CNN when using too deep network layers. When the network layer reaches a certain level and the network is too complicated, the accuracy rate will saturate and then decrease rapidly.

ResNet was proposed by He et al. in 2015 [42]. Because hierarchical networks have many redundancies, ResNet is designed to optimize network layer. The aim of ResNet is to complete the identity mapping and ensure that the input and output of the identity layer are the same. The identity layer of the network is determined automatically through training. ResNet changed several layers of the original network into a residual block.

**Figure 2:**
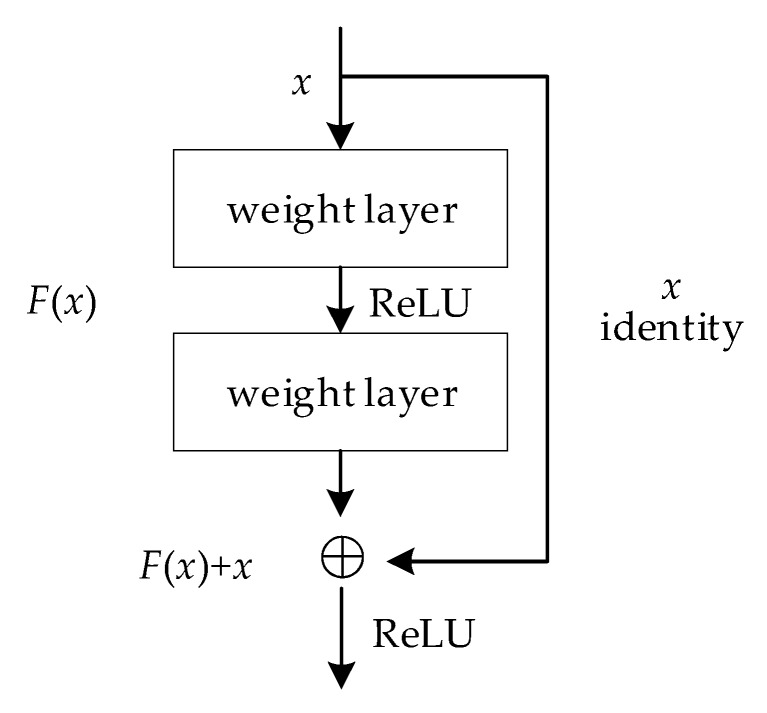
The identify block of ResNet.

The specific structure of the residual block is shown in Figure 2, where x is the input of this residual block and the residual is F(x). F(x) is the output after the linear transformation and the activation of the first layer. After the linear transformation of the second layer, the input x of this layer is added to F(x), and total activated by ReLU for getting output. The initial input x is added to the output of the second layer and then activated. This path is called shortcut connection. Establishing a direct correlation channel between the input and the output can make the parameterized layers focus on learning the residuals from the input to the output.

Residual operation is shown as Equations (6)–(8), where σ in Equation (6) represents the non-linear function ReLU. In Equation (7), y is the common output of the shortcut and the second ReLU. In Equation (8), when the input and output dimensions need to be changed, such as changing the number of channels, a linear transformation $W_{s}$ can be performed on x by the shortcut operation.

(6) $${F=W}_{2}{\sigma (W}_{2}x)$$

(7) $$y=F\left(x,\left\{W_{i}\right\}\right)+x$$

(8) $$y=F\left(x,\left\{W_{i}\right\}\right)+W_{s}x$$

## 4. ResCapNet for LiDAR Classification

**Figure 3:**
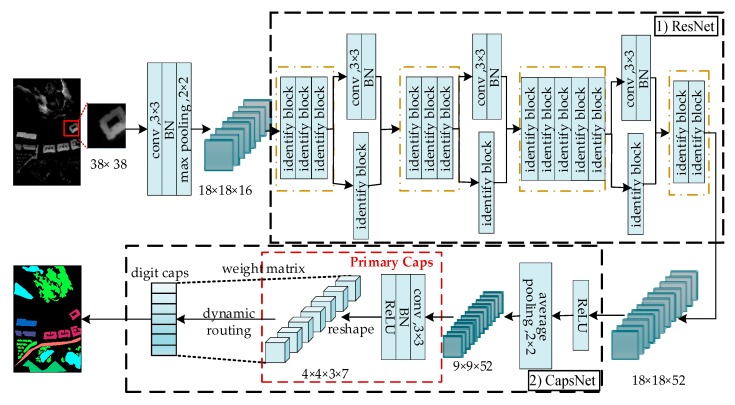
Architecture of the proposed method. The proposed architecture is composed of two subnetworks: 1) ResNet and 2) CapsNet. (1) The structure of the ResNet is modified based on ResNet-34 to make it suitable for LiDAR data sets. (2) The outputs of ResNet are sent to CapsNet for LiDAR classification.

The proposed method by us is shown as Figure 3. The network structure consists of two parts, the upper part is ResNet for extracting features and the lower part is CapsNet for classification.

### 4.1. Proposed Network Structure

**Figure 4:**
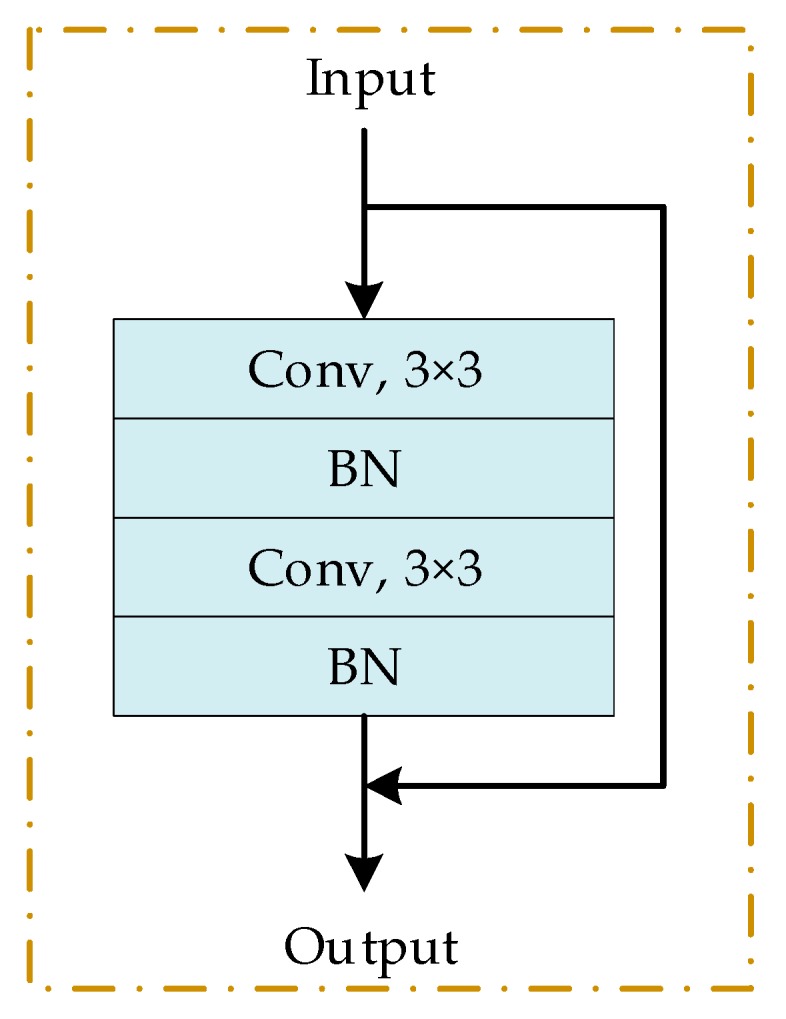
The identify block of ResNet used in this article.

We adopt the structure of ResNet34 and modify it to fit LiDAR data. Resnet-34 consists of four parts, each of which has three, four, six, and three identity blocks. Every identify block in each part has 64, 128, 256, and 512 filters, respectively. In the experiments of this article, because the size of the input is small, we reduced the size of the convolution kernel in the first convolution layer from 7 to 3 to ensure that the network can extract useful information. Meanwhile, reduce the number of filters used for each identify block in the four parts respectively to 16, 28, 40, and 52 and no output classification layer is used. Figure 4 shows the identity block used in this article, which consists of two convolutional layers and two batch normalization (BN) layers.

The parameter of dynamic routing in digit caps for the two data sets are all set to 3. The size of convolution kernel in primary caps is 3 × 3 and the channel is set to 3. Because there are seven land classes in Bayview Park data set, the number of vectors in primary caps and digit caps are both set to 7 and the number of capsules in digit caps is also set to 7. Meanwhile, there are 11 land classes in the Recology data set, so the number of vectors in primary caps and digit and the number of capsules in digit caps are all set to 11.

### 4.2. Adaptive Learning Optimization Algorithm

In this article, the Stochastic Gradient Descent (SGD) with momentum is used to back-propagate and update the network parameters for obtaining the optimal framework of ResCapNet, as shown in Equations (9) and (10),

(9) v=β·v−α·∇ω

(10) x←x+v

where α represents the learning rate and v represents the momentum factor. The gradient acts on v directly. When the direction of the negative gradient is the same with the direction of v, the direction of update is correct, and the weight will be updated quickly.

### 4.3. Loss and Activate Function

This article uses the ReLU function as the activation function of the network. In Equation (11), some outputs of the neuron are set to zero, which can reduce the dependency between the parameters and alleviate the overfitting phenomenon of the network.

(11) g(x)=max(0,x)

We adopt the softmax function to classify and choose the exponential form of softmax in Equation (12).

(12) $$a_{j}^{L}=\frac{e^{\left(Z_{j}^{L}\right)}}{\sum_{K}e^{\left(Z_{K}^{L}\right)}}$$

The input of the last layer is $Z_{j}^{L}$, the output of the last layer is $a_{j}^{L}$ and e is a constant. The inputs of all neurons in the $L^{th}$ layer is $\sum_{K}e^{\left(Z_{K}^{L}\right)}$. Therefore, the loss function is cross-entropy loss in Equation (13).

(13) $${\mathrm{Loss}}_{i}=-\log y_{i}=-\log\frac{e^{\left(Z_{j}^{L}\right)}}{\sum_{K}e^{\left(Z_{K}^{L}\right)}}$$

## 5. Experimental Results and Analysis

### 5.1. Algorithm Data Description

**Figure 5:**
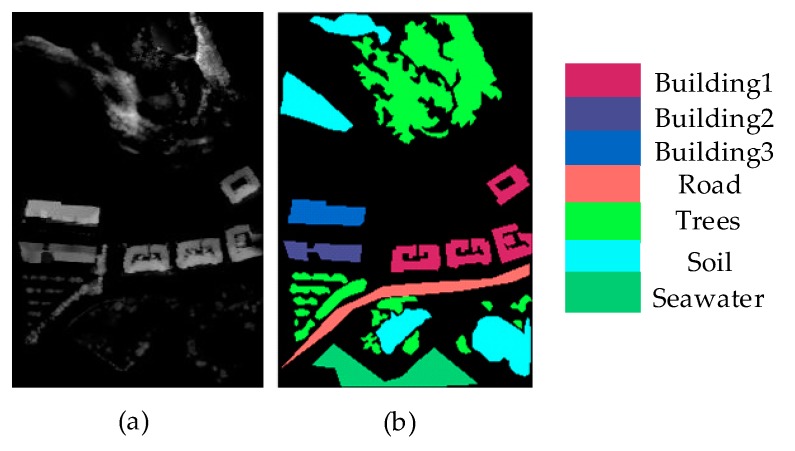
Bayview Park data set: (**a**) DSM map; (**b**) Groundtruth map.

In this article, two different LiDAR data sets were used to evaluate the proposed method; one is Bayview Park data set and the other is Recology data set. They were obtained from the 2012 IEEE International Remote Sensing Image Convergence Competition. The Bayview Park data set was collected in June 2010 by the sensor WorldView2 in San Francisco, USA, as shown in Figure 5. The data set had a spatial resolution of 1.8m and contains 300 × 200 pixels. It had seven land classes, which were building1, building2, building3, road, trees, soil, and seawater.

**Figure 6:**
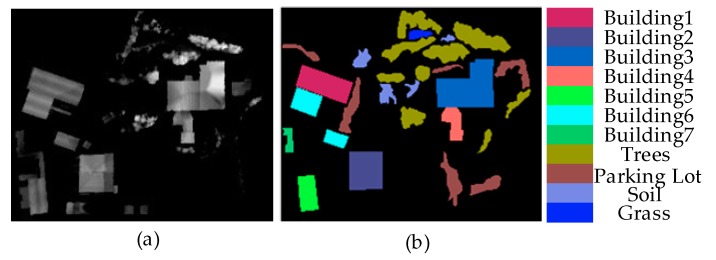
Recology data set: (**a**) DSM map; (**b**) Groundtruth map.

Figure 6. shows the Recology data set, which was also acquired in an urban location in San Francisco, USA. It contained 200 × 250 pixels and had a spatial resolution of 1.8 m. It had 11 land classes, which were building1, building2, building3, building4, building5, building6, building6, trees, parking lot, soil, and grass.

### 5.2. Experimental Setup

The experiments in this article were carried out under Windows system and accelerated with Nvidia RTX2060(Asus, Taiwan, China) graphics card. The codes take tensorflow as the backend and are implemented through the Keras and the python (Anaconda, Austin, Texas). The data sets were divided into training sets and test sets. We selected 400, 500, 600, and 700 samples randomly in the data sets as the training set, and the rest for testing the effect of the model. Verified by experiments, it was better to set the size of the input for ResCapNet to 38 × 38 pixels, meanwhile the input size of all comparative experiments was set to 38 × 38 pixels, and the DSM data were linearly mapped to [−0.5, 0.5]. The training batch size of the data sets was 32. Set 150 epochs for training, and when the classification accuracy of the network no longer increases (exceeding 20 epochs), the training will stop early. Selecting the ‘same’ for the fill pattern of each layer’s feature maps, so that the length and width of each layer’s inputs and outputs are unchanged. The structure of CNN is shown in Table 1.

We use SGD algorithm with momentum as the gradient optimizer. The momentum was selected to 0.9 and the descent rate was selected to 10^−6^. When training the ResCapNet model, the initial learning rate for the Bayview Park data set and the Recology data set were set to 0.001, and when training the CNN and the ResNet models, the initial learning rate were also set to 0.001. For the Bayview Park data set, the maximum depth of the decision tree was set to 100, and for the Recology data set, the maximum depth of the decision tree was set to 25. The kernel function of the SVM was set to the radial basis function (rbf), the rbf coefficient defaults to “auto”, and the penalty parameter of the error term was set to 100. The value of k for the KNN was set to 1, the leaf_size was set to 30, and the metric distance select to Euclidean distance. The estimates of the Random Forest for the two data sets were set to 30.

### 5.3. Experimental Results and Aanlysis

We adopted overall accuracy (OA), average accuracy (AA), kappa coefficient (K), recall, precision, and RGB false color map to evaluate the performance of the model. Table 2 and Table 3 provide the classification results of different methods for Bayview Park data set and Recology data set when selecting 400, 500, 600, and 700 training samples, respectively.

We can see that ResCapNet always achieved the highest accuracy and the best OA were 96.12% ± 0.51% for the Bayview Park data set and 96.39% ± 0.79% for the Recology data set. The best OA of Bayview Park data set was 0.70%, 1.33%, 5.95%, 5.51%, 5.69%, 10.06%, 18.91%, and 19.27% higher than OctSqueezeNet, ResNet, CapsNet, CNN, Random Forest, KNN, SVM, and Decision Tree, respectively. The best OA of Recology data set increased 0.48%, 0.67%, 6.22%, 3.91%, 4.68%, 8.03%, 19.18%, and 20.09% compared to OctSqueezeNet, ResNet, CapsNet, CNN, Random Forest, KNN, SVM, and Decision Tree, respectively.

**Figure 7:**
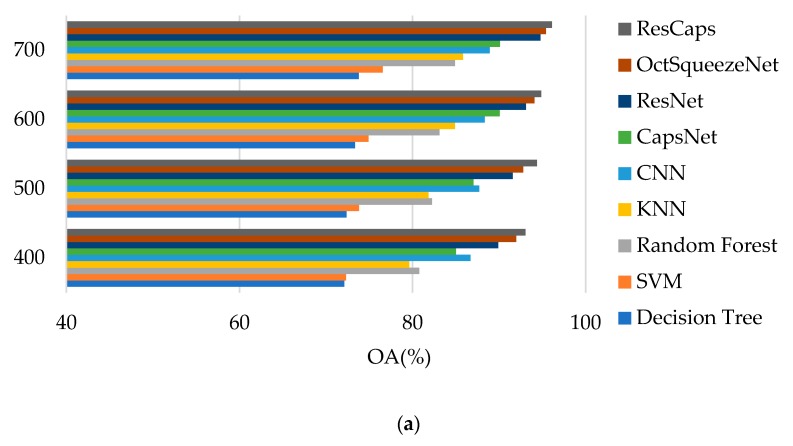

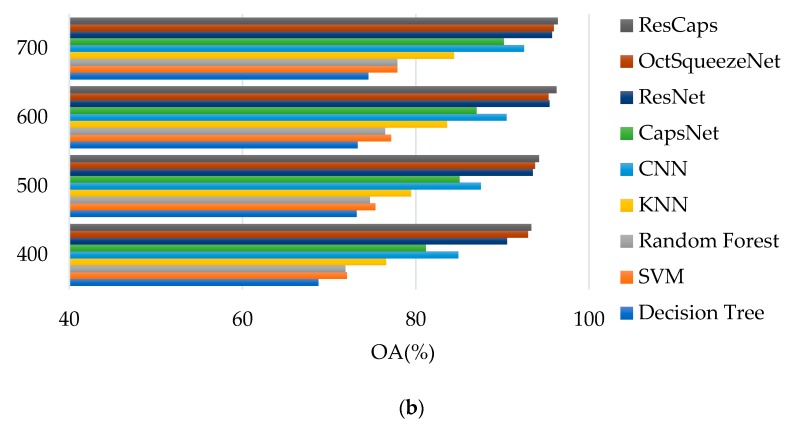
Classification results of different methods: (**a**) Bayview Park data set; (**b**) Recology data set.

Figure 7 is a comparison of the test results of different methods when 700 training samples were selected for the two data sets. It can be intuitively seen that the method proposed by us had the best classification effect. Table 4 and Table 5 give the precision and recall of each class for 700 samples on Bayview Park data set and Recology data set. Table 6 and Table 7 give the classification accuracy of per class on Bayview Park data set and Recology data set. According to the classification results of each land classes shown in these four tables, when CapsNet was used alone, the classification effect of land classes with lower height was good, because it was sensitive to spatial features, but its overall classification accuracy was not high. When ResNet was used alone, the classification accuracy of land classes with higher height was high, but it was difficult to identify the land classes with lower height. The combination of the two greatly reduced the influence for the height of the land classes on the classification results, and the classification accuracy of each category was very high.

**Figure 8:**
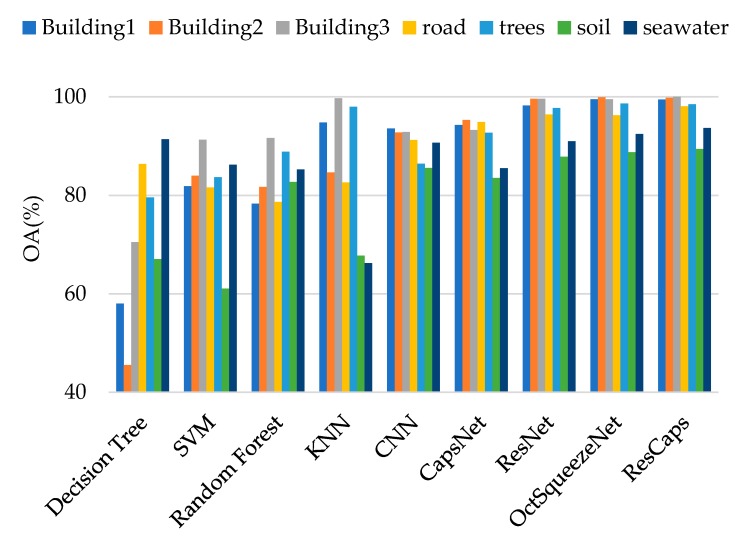
Classification results of different methods for each class on Bayview Park.

**Figure 9:**
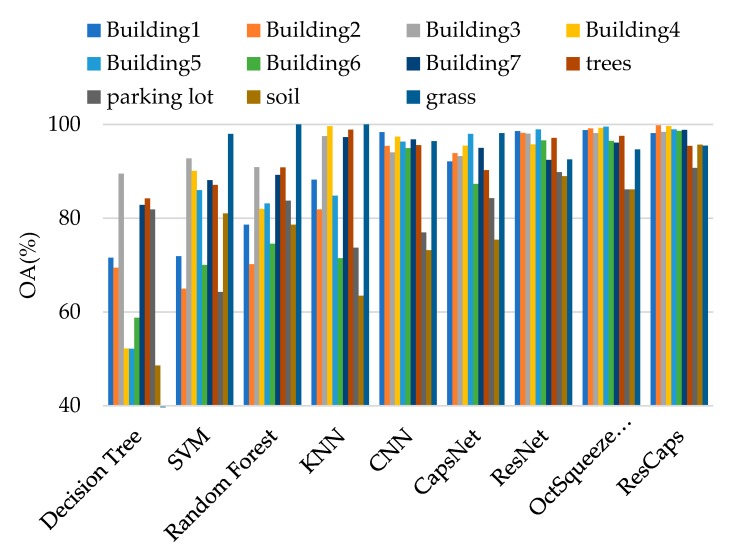
Classification results of different methods for each class on Recology data set.

**Figure 10:**
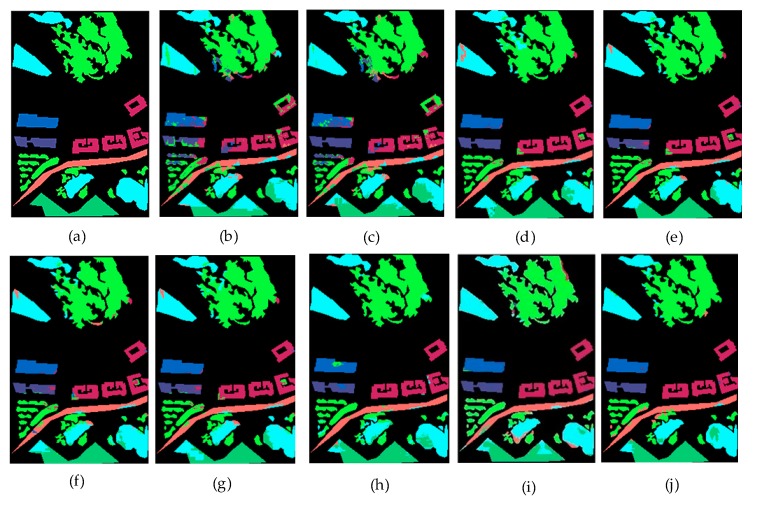
Classification results on Bayview Park data set: (**a**) Ground-truth map; (**b**) Decision Tree; (**c**) SVM; (**d**) KNN; (**e**) Random Forest; (**f**) CNN; (**g**) CapsNet; (**h**) ResNet; (**i**) OctSqueezeNet; (**j**) ResCapNet.

**Figure 11:**
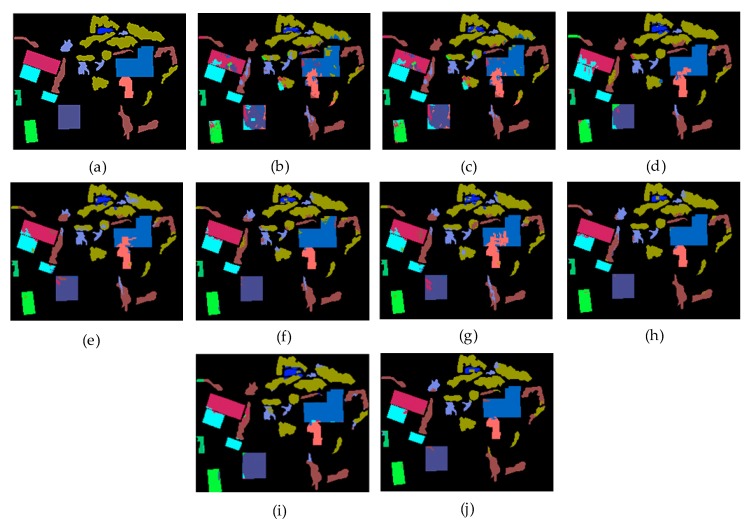
Classification results on Recology data set: (**a**) Ground-truth map; (**b**) Decision Tree; (**c**) SVM; (**d**) KNN; (**e**) Random Forest; (**f**) CNN; (**g**) CapsNet; (**h**) ResNet; (**i**) OctSqueezeNet; (**j**) ResCapNet.

Figure 8 and Figure 9 visually show the classification results of each class on the two data sets. It can be clearly seen that classification results of ResCapNet for each class were excellent. Figure 10 and Figure 11 provides classification maps for different classifiers.

## 6. Conclusions

This article designs a deep learning model-ResCapNet, which combines the advantages of ResNet and CapsNet for improving the original structure to effectively classify remotely sensed LiDAR data. The two well-known LiDAR data sets are considered in this article, and eight established algorithms are used to compare with our proposed method, it can be seen that, competitive with state-of-the-art classification methods for LiDAR, our proposed method can achieve better classification results. It achieves 96.12% and 96.39% in terms of OA on the Bayview Park and Recology data sets, respectively, when the number of training samples is selected 700.

The shortcut channel of ResNet can retain more complete feature information and alleviate the problem of network performance degradation caused by the inappropriate depth of CNN. At the same time, it automatically extracts effective features from the data. This enables subsequent CapsNet to learn more useful feature information. Meanwhile, because the sensitivity of CapsNet to space transformation of features, it can extract more detailed feature information and retain more valuable information compared to ordinary CNNs. Thus, the combination of the two structure obtains a very good classification effect.

In addition, the practical effects of this methods on other remote sensing data sets need to be continuously verified. Meanwhile, we need to further explore how to automatically generate an optimal network model suitable for LiDAR classification.

## Figures and Tables

**Table 1:** Architecture of CNN.

NO.	Conv	ReLU	Pool	Stride
1	3 × 3 × 1 × 20	Yes	2 × 2	1
2	3 × 3 × 20 × 20	Yes	2 ×2	1

**Table 2:** Classification results of different training samples on Bayview Park data set.

	Training Samples	Index	400	500	600	700
Methods						
Decision Tree		OA%	76.84 ± 0.51	76.46 ± 0.71	76.66 ± 1.53	76.85 ± 1.55
		AA%	71.24 ± 1.43	71.80 ± 2.31	72.04 ± 2.29	72.23 ± 3.14
		K×100	68.04 ± 1.69	68.35 ± 1.21	67.71 ± 2.11	69.73 ± 0.60
SVM		OA%	72.48 ± 2.12	76.79 ± 0.31	76.91 ± 2.01	77.21 ± 0.88
		AA%	76.87 ± 1.42	78.59 ± 1.97	78.85 ± 1.15	81.19 ± 2.31
		K×100	67.32 ± 1.69	68.39 ± 1.04	68.82 ± 1.67	69.81 ± 2.33
KNN		OA%	79.51 ± 0.27	81.90 ± 0.38	85.25 ± 0.19	86.06 ± 0.77
		AA%	81.35 ± 0.16	83.42 ± 0.06	84.92 ± 0.82	87.47 ± 0.37
		K×100	73.80 ± 0.22	76.49 ± 0.37	79.94 ± 0.35	81.95 ± 0.36
Random Forest		OA%	86.78 ± 0.40	87.75 ± 0.31	88.16 ± 0.44	90.43 ± 0.67
		AA%	88.75 ± 1.74	89.20 ± 0.17	89.33 ± 0.48	89.95 ± 0.95
		K×100	82.33 ± 0.62	83.61 ± 0.38	84.06 ± 0.59	86.57 ± 0.87
CNN		OA%	87.35 ± 1.91	87.91 ± 1.16	88.33 ± 0.73	90.61 ± 1.89
		AA%	88.90 ± 1.03	89.63 ± 2.71	89.51 ± 2.04	90.23 ± 0.68
		K×100	82.72 ± 1.67	85.02 ± 1.85	86.03 ± 1.98	86.72 ± 2.34
CapsNet		OA%	85.01 ± 1.47	87.05 ± 1.19	90.07 ± 1.18	90.11 ± 0.91
		AA%	83.89 ± 2.13	87.78 ± 1.70	91.34 ± 1.24	91.64 ± 1.73
		K×100	80.21 ± 1.81	82.85 ± 0.79	86.81 ± 1.45	86.92 ± 1.22
ResNet		OA%	89.91 ± 2.07	91.57 ± 1.76	93.12 ± 1.51	94.79 ± 0.90
		AA%	91.03 ± 1.88	93.23 ± 0.81	94.25 ± 1.06	95.78 ± 1.34
		K×100	86.62 ± 1.99	88.84 ± 2.39	90.91 ± 2.07	93.53 ± 1.17
OctSqueezeNet		OA%	91.99 ± 0.81	92.79 ± 0.41	94.09 ± 1.23	95.42 ± 0.91
		AA%	93.21 ± 0.43	95.02 ± 0.90	95.75 ± 1.25	96.43 ± 1.37
		K×100	89.48 ± 1.00	90.48 ± 0.47	92.23 ± 1.64	93.99 ± 1.97
ResCapNet		OA%	93.05 ± 0.63	94.39 ± 0.57	94.87 ± 0.56	96.12 ± 0.51
		AA%	94.36 ± 0.84	95.45 ± 0.79	96.03 ± 0.76	97.01 ± 1.09
		K×100	90.77 ± 0.98	92.56 ± 0.53	93.22 ± 0.77	94.89 ± 1.14

**Table 3:** Classification results of different training samples on Recology data set.

	Training Samples	Index	400	500	600	700
Methods						
Decision Tree		OA%	68.73 ± 1.22	73.08 ± 0.13	74.11 ± 0.28	76.30 ± 0.29
		AA%	60.49 ± 2.02	64.28 ± 1.35	66.27 ± 0.62	68.58 ± 1.37
		K×100	63.01 ± 1.40	68.10 ± 0.01	69.38 ± 0.32	70.06 ± 0.33
SVM		OA%	72.48 ± 2.12	76.79 ± 0.31	76.91 ± 2.01	77.23 ± 0.88
		AA%	76.87 ± 1.42	78.59 ± 1.97	78.85 ± 1.15	81.19 ± 2.31
		K×100	67.32 ± 1.69	68.39 ± 1.04	68.82 ± 1.67	69.81 ± 2.33
KNN		OA%	77.62 ± 0.82	84.73 ± 0.16	85.58 ± 0.03	88.36 ± 1.24
		AA%	80.29 ± 0.98	85.78 ± 2.98	85.31 ± 0.40	89.27 ± 1.05
		K×100	73.54 ± 0.76	80.29 ± 0.12	83.08 ± 0.08	86.29 ± 1.04
Random Forest		OA%	85.17 ± 1.35	87.22 ± 0.83	88.79 ± 2.07	91.71 ± 1.02
		AA%	88.19 ± 2.13	89.85 ± 3.06	90.01 ± 1.45	91.15 ± 1.43
		K×100	82.16 ± 0.76	86.26 ± 1.57	86.54 ± 2.11	89.01 ± 1.22
CNN		OA%	85.91 ± 1.33	88.51 ± 1.22	90.47 ± 0.62	92.48 ± 1.69
		AA%	88.46 ± 2.36	90.36 ± 0.43	90.31 ± 1.04	92.07 ± 1.95
		K×100	83.03 ± 1.51	87.08 ± 0.79	86.67 ± 0.77	89.96 ± 1.80
CapsNet		OA%	81.17 ± 1.46	85.04 ± 1.73	87.02 ± 0.84	90.17 ± 1.18
		AA%	82.75 ± 2.34	86.82 ± 1.44	87.62 ± 1.60	91.17 ± 1.87
		K×100	77.43 ± 1.89	82.13 ± 1.02	84.56 ± 1.03	88.23 ± 1.43
ResNet		OA%	90.53 ± 1.83	93.51 ± 1.39	95.43 ± 0.66	95.72 ± 0.95
		AA%	88.70 ± 2.08	94.47 ± 1.13	94.28 ± 1.25	95.16 ± 1.75
		K×100	88.77 ± 2.33	92.94 ± 1.68	94.92 ± 0.79	95.06 ± 1.14
OctSqueezeNet		OA%	92.94 ± 0.21	93.75 ± 1.23	95.07 ± 0.48	95.91 ± 0.73
		AA%	93.63 ± 0.17	93.72 ± 0.60	95.36 ± 1.15	95.89 ± 0.17
		K×100	92.79 ± 0.74	93.79 ± 0.99	94.13 ± 0.63	95.13 ± 0.11
ResCapNet		OA%	93.34 ± 1.22	94.21 ± 1.24	96.23 ± 0.98	96.39 ± 0.79
		AA%	94.25 ± 0.81	95.27 ± 0.42	97.16 ± 1.05	97.31 ± 1.02
		K×100	91.17 ± 0.80	93.10 ± 1.03	95.51 ± 0.88	95.70 ± 0.65

**Table 4:** Precision and recall of each class for 700 samples on Bayview Park data set.

**precision**		**Classes**	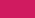	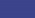	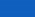	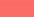	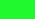	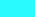	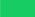
	**Methods**								
	**Decision** **Tree**		**0.59**	**0.52**	**0.83**	**0.76**	**0.88**	**0.81**	**0.62**
	**SVM**		**0.83**	**0.80**	**0.78**	**0.80**	**0.84**	**0.61**	**0.88**
	**KNN**		**0.98**	**0.77**	**0.97**	**0.82**	**0.99**	**0.70**	**0.70**
	**Random** **Forest**		**0.84**	**0.94**	**1.00**	**1.00**	**0.91**	**0.82**	**0.89**
	**CNN**		**0.99**	**0.87**	**0.87**	**0.94**	**1.00**	**0.78**	**0.87**
	**CapsNet**		**0.93**	**0.98**	**0.86**	**0.98**	**0.92**	**0.85**	**0.79**
	**ResNet**		**0.97**	**1.00**	**1.00**	**0.86**	**0.97**	**0.90**	**0.82**
	**OctSqueezeNet**		**1.00**	**0.99**	**0.98**	**0.92**	**0.99**	**0.87**	**0.89**
	**ResCapNet**		**1.00**	**1.00**	**1.00**	**0.97**	**1.00**	**0.96**	**0.93**
recall		classes	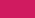	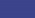	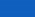	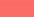	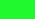	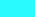	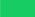
	methods								
	Decision Tree		0.70	0.74	0.78	0.66	0.81	0.79	0.72
	SVM		0.79	0.73	0.90	0.46	0.77	0.92	0.52
	KNN		0.95	0.96	0.96	0.87	0.76	0.94	0.74
	Random Forest		0.93	0.42	0.91	0.71	0.98	0.93	0.70
	CNN		0.96	0.85	0.94	0.80	0.94	0.99	0.66
	CapsNet		0.85	0.63	0.96	0.78	0.99	0.88	0.79
	ResNet		0.96	0.99	0.98	0.94	0.98	0.86	0.84
	OctSqueezeNet		0.99	0.99	1.00	0.93	0.95	0.95	0.86
	ResCapNet		**0.99**	**1.00**	**1.00**	**0.98**	**0.99**	**0.97**	**0.93**

**Table 5:** Precision and recall of each class for 700 samples on Recology data set.

**precision**		**Classes**	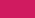	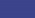	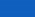	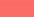	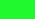	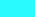	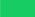	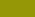	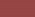	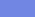	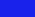
	**Methods**												
	**Decision** **Tree**		**0.74**	**0.59**	**0.88**	**0.76**	**0.69**	**0.61**	**0.55**	**0.87**	**0.87**	**0.51**	**0.29**
	**SVM**		**0.74**	**0.78**	**0.96**	**0.91**	**0.77**	**0.77**	**0.84**	**0.86**	**0.65**	**0.76**	**1.00**
	**KNN**		**0.88**	**0.88**	**0.98**	**0.96**	**0.89**	**0.76**	**0.93**	**0.99**	**0.68**	**0.36**	**1.00**
	**Random** **Forest**		**0.98**	**0.92**	**0.88**	**1.00**	**0.97**	**0.98**	**1.00**	**0.86**	**0.86**	**0.81**	**1.00**
	**CNN**		**0.99**	**0.99**	**0.97**	**0.92**	**0.94**	**0.89**	**0.84**	**0.96**	**0.83**	**0.86**	**0.88**
	**CapsNet**		**0.82**	**0.87**	**0.95**	**0.95**	**0.97**	**0.89**	**0.94**	**0.92**	**0.90**	**0.83**	**0.85**
	**ResNet**		**0.98**	**0.99**	**0.98**	**0.99**	**1.00**	**0.98**	**0.95**	**0.98**	**0.91**	**0.90**	**0.95**
	**OctSqueezeNet**		**0.99**	**1.00**	**1.00**	**1.00**	**1.00**	**0.98**	**1.00**	**0.99**	**0.88**	**0.90**	**1.00**
	**ResCapNet**		**0.99**	**1.00**	**0.97**	**1.00**	**0.99**	**1.00**	**1.00**	**0.98**	**0.93**	**0.98**	**0.96**
recall		classes	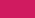	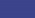	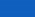	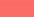	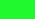	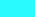	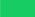	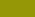	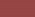	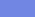	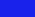
	methods												
	Decision Tree		0.63	0.76	0.84	0.51	0.79	0.56	0.93	0.84	0.84	0.58	0.33
	SVM		0.83	0.69	0.96	0.89	0.71	0.65	0.60	0.87	0.92	0.11	0.17
	KNN		0.97	0.89	0.94	0.86	0.94	0.91	0.96	0.80	0.68	0.72	0.54
	Random Forest		0.91	0.92	0.98	0.53	0.92	0.71	0.98	1.00	1.00	0.32	0.23
	CNN		0.99	0.99	0.97	0.92	0.94	0.89	0.84	0.96	0.83	**0.86**	0.88
	CapsNet		0.97	0.85	0.94	0.64	0.93	0.84	1.00	0.97	0.91	0.52	0.82
	ResNet		0.99	1.00	0.99	0.96	1.00	0.96	0.92	0.99	0.97	0.71	**0.88**
	OctSqueezeNet		0.99	1.00	1.00	**0.99**	0.99	**0.99**	0.75	1.00	0.97	0.67	0.94
	ResCapNet		**0.99**	**1.00**	**1.00**	0.92	**0.99**	0.98	**0.96**	**1.00**	**0.98**	0.73	0.87

**Table 6:** Classification results of each class for 700 samples on Bayview Park data set.

Classes	Decision Tree	SVM	KNN	Random Forest	CNN	CapsNet	Res-Net	OctSque-ezeNet	Res-CapNet
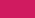	68.08 ± 5.13	81.88 ± 3.91	99.50 ± 1.06	95.18 ± 3.89	93.58 ± 1.53	94.31 ± 1.47	98.25 ± 1.55	99.52 ± 0.09	**99.47 ± 0.53**
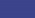	53.69 ± 9.28	84.01 ± 3.12	80.88 ± 1.89	98.81 ± 1.20	92.78 ± 1.12	95.32 ± 2.13	99.62 ± 0.38	**99.93 ± 0.07**	99.82 ± 0.18
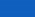	73.01 ± 4.49	91.31 ± 5.04	**100**	**100**	92.87 ± 1.48	93.26 ± 1.81	99.60 ± 2.86	99.54 ± 0.46	**100**
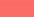	72.56 ± 0.12	81.60 ± 4.43	90.84 ± 2.66	82.55 ± 6.37	91.25 ± 1.47	94.88 ± 1.19	96.43 ± 2.77	96.29 ± 2.77	**98.12 ± 1.22**
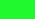	86.68 ± 2.29	83.67 ± 1.86	98.15 ± 0.31	90.48 ± 1.16	86.43 ± 1.61	92.74 ± 1.70	97.72 ± 0.93	**98.67 ± 0.88**	98.52 ± 0.60
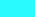	78.43 ± 5.27	61.04 ± 3.46	70.62 ± 1.09	87.02 ± 0.57	85.57 ± 1.69	83.53 ± 0.79	87.87 ± 2.11	88.75 ± 3.26	**89.44 ± 2.63**
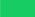	66.10 ± 0.46	86.23 ± 2.92	72.26 ± 0.43	84.03 ± 0.91	90.69 ± 2.68	85.51 ± 1.22	90.99 ± 2.76	92.47 ± 2.30	**93.68 ± 2.45**

**Table 7:** Classification results of each class for 700 samples on Recology data set.

Classes	Decision Tree	SVM	KNN	Random Forest	CNN	CapsNet	Res-Net	OctSque-ezeNet	Res-CapNet
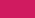	71.87 ± 4.84	71.87 ± 1.01	90.66 ± 4.99	91.04 ± 3.59	98.34 ± 1.19	92.09 ± 1.09	98.54 ± 1.46	**99.06 ± 0.94**	98.13 ± 1.60
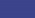	67.46 ± 2.29	64.97 ± 1.94	82.26 ± 4.27	95.40 ± 4.71	95.40 ± 1.36	93.86 ± 1.21	98.17 ± 1.83	99.56 ± 0.44	**99.76 ± 0.24**
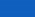	83.85 ± 3.04	92.74 ± 1.10	95.07 ± 1.84	93.99 ± 1.49	93.99 ± 1.07	93.21 ± 1.12	98.03 ± 1.97	98.12 ± 1.43	**98.41 ± 1.16**
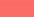	61.09 ± 1.44	90.05 ± 2.11	96.38 ± 0.67	97.35 ± 0.35	97.35 ± 1.24	95.46 ± 1.13	95.71 ± 1.86	99.55 ± 0.45	**99.63 ± 0.37**
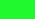	66.72 ± 1.12	85.98 ± 1.42	91.53 ± 3.04	96.30 ± 2.77	96.30 ± 2.02	97.96 ± 1.95	**98.92 ± 1.08**	**100**	98.90 ± 1.10
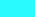	48.55 ± 6.93	70.04 ± 0.81	89.26 ± 1.38	94.91 ± 1.24	94.91 ± 1.18	87.28 ± 1.41	96.56 ± 2.29	95.35 ± 1.81	**98.58 ± 1.42**
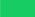	70.22 ± 9.40	88.09 ± 2.98	86.59 ± 2.68	96.78 ± 2.88	96.78 ± 1.87	95.00 ± 1.79	92.43 ± 2.53	96.94 ± 1.64	**98.81 ± 1.19**
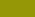	87.54 ± 2.85	87.05 ± 1.30	87.88 ± 1.55	95.57 ± 0.18	95.57 ± 1.16	90.22 ± 0.61	97.11 ± 1.62	**97.54 ± 2.01**	95.41 ± 1.21
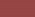	80.76 ± 1.41	64.26 ± 1.71	87.34 ± 1.02	76.94 ± 0.06	76.94 ± 1.27	84.29 ± 0.79	89.80 ± 2.03	87.48 ± 1.27	**90.72 ± 1.76**
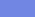	52.37 ± 0.93	81.03 ± 3.99	80.25 ± 1.30	73.16 ± 0.32	73.16 ± 1.51	75.42 ± 1.46	88.97 ± 2.67	89.68 ± 0.53	**95.68 ± 2.00**
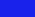	54.77 ± 3.34	**97.94 ± 1.48**	91.63 ± 1.24	98.13 ± 1.33	96.43 ± 1.41	98.13 ± 1.27	92.54 ± 2.46	91.68 ± 1.61	95.47 ± 2.48
